# The complementary roles of genome-wide approaches in identifying genes linked to an inherited risk of colorectal cancer

**DOI:** 10.1186/s13053-023-00245-5

**Published:** 2023-01-28

**Authors:** Olfat Ahmad, Asta Försti

**Affiliations:** 1 Hopp Children’s Cancer Center (KiTZ), Heidelberg, Germany; 2 Division of Pediatric Neurooncology, German Cancer Research Center (DKFZ), German Cancer Consortium (DKTK), Heidelberg, Germany; 3 Institute of Human Genetics, University Hospital Heidelberg, Heidelberg, Germany; 4 University of Oxford, Oxford, UK; 5 King Hussein Cancer Center (KHCC), Amman, Jordan

**Keywords:** CRC, GWAS, WGS, High-risk gene, Low-risk gene, Genome-wide, Inherited Cancer, Cancer predisposition

## Abstract

The current understanding of the inherited risk of colorectal cancer (CRC) started with an observational clinical era in the late 19^th^ century, which was followed by a genetic era starting in the late 20^th^ century. Genome-wide linkage analysis allowed mapping several high-risk genes, which marked the beginning of the genetic era. The current high-throughput genomic phase includes genome-wide association study (GWAS) and genome-wide sequencing approaches which have revolutionized the conception of the inherited risk of CRC. On the one hand, GWAS has allowed the identification of multiple low risk loci correlated with CRC. On the other, genome-wide sequencing has led to the discovery of a second batch of high-to-moderate-risk genes that correlate to atypical familial CRC and polyposis syndromes. In contrast to other common cancers, which are usually dominated by a polygenic background, CRC risk is believed to be equally explained by monogenic and polygenic architectures, which jointly contribute to a quarter of familial clustering. Despite the fact that genome-wide approaches have allowed the identification of a continuum of responsible high-to-moderate-to-low-risk variants, much of the predisposition and familial clustering of CRC has not yet been explained. Other genetic, epigenetic and environmental factors might be playing important roles as well. In this review we aim to provide insights on the complementary roles played by different genomic approaches in allowing the current understanding of the genetic architecture of inherited CRC.

## Introduction

Familial clustering of colorectal cancer (CRC) and gynecological tumors in families affected with what later came to be identified as Lynch syndrome (LS) was among the first clinical clues that led to the proposal of the principle of “inherited cancer” in the late 19^th^ century. Back then, the idea was completely eccentric, and was first addressed in 1866 by the neuroanatomist Paul Broca who presented the clustering of 15 cases of breast cancer in his wife’s family [1]. In 1895, the pathologist Aldred Warthin “officially” proposed the concept of cancer predisposition by establishing a 4-generation pedigree for “family G” who had CRC and gynecological tumors transmitted in an autosomal dominant (AD) fashion [2]. An update about the family was published in the mid-1930s by Warthin’s colleagues Hauser and Weller. Yet, no specific diagnosis was made [2]. In the meantime, familial adenomatous polyposis (FAP) was starting to come to light. In 1925, the senior surgeon Percy Lockhart-Mummery, described 3 families with polyposis and CRCs at an early age. In 1939, Cuthbert E. Dukes, together with Lockhart-Mummery, published 7 other families showing the same AD inheritance, and they contributed the condition to an inherited abnormality [3]. In 1962, during his internal medicine residency, Henry Lynch came across a CRC patient with a family history similar to “family G” without evidence of polyposis, and in 1966, published the pedigree of the family together with another family identified by Marjorie Shaw [2]. Since then, international collaborations have been initiated by Lynch and colleagues with the aim of collecting and evaluating cancer-prone families [4], and it was not until 1984 that the terminologies of hereditary non-polyposis colorectal cancer (HNPCC) and LS were coined [2].

During the late 1980s, the molecular basis of familial cancers began to be unraveled. In 1986, *RB1* was the first identified cancer predisposition gene (CPG) [5, 6]. *APC* was identified in 1991 [7], and *MSH2* was the first identified LS locus in 1993, when microsatellite instability (MSI) was first described as well [2]. Linkage analysis is the classic method which has contributed to the discovery of more than a 100 high-to-moderate risk CPGs [1, 8], including the major CRC risk genes [9–11]. During this era, it became known that 2–8% of CRCs could be attributed to inherited genetic defects. However, despite the great contribution of linkage analysis to CPG mapping, the number of identified genes had soon come to a plateau, and the focus shifted toward a polygenic model of inheritance. In 2005, the first GWAS for a non-cancerous disease was published, and since then more than 50,000 associations have been reported, which have revolutionized the understanding of genetic architecture of several inherited diseases, including cancer [12–14]. As for CRC, GWASs have identified ~ 100 validated loci accounting for ∼12% of familial relative risk [15], and have additionally offered a possible explanation for the inherited personalized risk of CRC when the family history is negative [11]. However, as the rare high-to-moderate penetrant variants and the common variants with ultra-small effect sizes are not well interrogated by GWASs, many questions regarding CRC inheritance were left unanswered [16, 17]. In the last 10 years, genome-wide sequencing approaches have led to the identification of a second batch of rare CRC genes that are mainly of moderate-risk. These genes have helped define new familial CRC and polyposis syndromes [11].

**Fig. 1:**
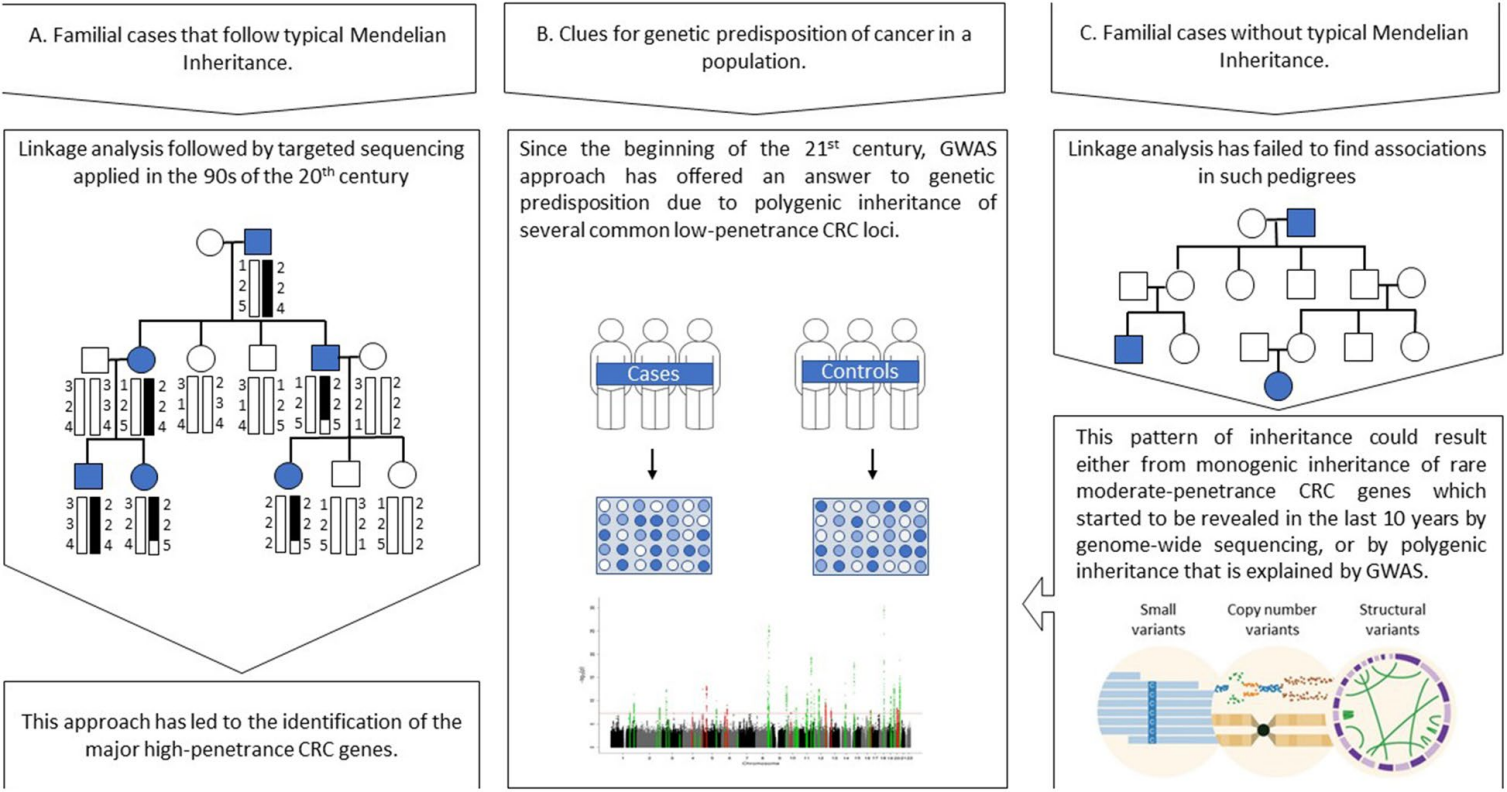
Summary of the genome-wide approaches utilized to identify CRC risk genes

Together, genome-wide approaches have aided the current understanding of inherited risk of CRC, and it is believed nowadays that contrary to most common cancers which are dominated by polygenic architecture, CRC is equally governed by monogenic and polygenic inheritance [11]. This review aims at highlighting the complementary roles played by different genomic approaches that have led to the current knowledge about the genetic architecture of inherited CRC (Fig. 1).

## Linkage analysis: the classical high-penetrance CRC genes

Monogenic inheritance refers to a type of Mendelian inheritance whereby a trait is determined by the expression of a single high-risk gene. This term is related to the phrase “rare allele model of inheritance”, as it attributes inherited diseases to the inheritance of any of various rare deleterious alleles contributing to the disease. Each of these rare variants occurs at a frequency of < 1% and follows a monogenic inheritance architecture in the sense of elevating the risk to two-fold or more against the background [1, 17]. Classically, there used to be multiple arguments supporting this model of inheritance, of which the evolutionary theory provides the strongest ground by advocating that deleterious variants should be selected against, and hence could not be common [17]. Further supporting this model of inheritance are the multiple familial disorders which can be browsed on the Online Mendelian Inheritance in Man (OMIM) database [18].

Genome-wide linkage analysis, which was developed in the second half of the 20^th^ century, is the classic statistical method applied for mapping Mendelian traits to their chromosomal locations by examining genome-wide markers such as microsatellite markers (and later on, single nucleotide polymorphisms (SNPs)) in pedigrees segregating the trait. After identifying the chromosomal region linked to the trait, targeted sequencing is conducted to identify the particular responsible gene (Fig. 1A). An accurate extended pedigree with a near-typical Mendelian inheritance pattern is an important prerequisite for linkage analysis to yield a statistically significant result [16].

As for CRC, linkage analysis has allowed the identification of the 10 classic high-penetrance genes that contribute to the well-known hereditary CRC and polyposis syndromes. These genes include the 4 DNA mismatch repair (MMR) genes, *MLH1, MSH2, MSH6* and *PMS2,* that are inherited in an AD manner and cause LS when mutated. There are also the *APC* gene with AD mutations, and the *MUTYH* gene, which follow autosomal recessive (AR) inheritance, and contribute to FAP, together with *SMAD4, BMPR1A, STK11* and *PTEN* which contribute to the less frequent CRC predisposing syndromes characterized by the presence of hamartomatous polyps [10, 11]. These discoveries of hereditary CRC and polyposis syndromes, together with the development of multiple-CPG panels [10] have led to significant implications on clinical oncology practice, facilitating cancer predisposition services [19]. In addition, patients with some CRC predisposition syndromes can particularly benefit from therapeutic options such as immunotherapy for LS patients [20].

On the other hand, there are two major problems that have come to light with the discoveries of these high-penetrance CPGs. The first is the psychosocial burden of available genetic information on carrier families who need to adapt to the dilemma of living with the uncertainties of several family members manifesting the various tumors predicted by the genetic testing. An *MSH2* carrier for example has an 80.4% probability that he or she will develop any cancer by the age of 75 years, including a 43% risk of CRC and a 65.7% risk of a gynecological cancer for women, in addition to a 50% probability that each of their offspring carries the variant with its risks of various malignancies [21]. The second concern is that much of the familial cases have not been solved by these genes. This gap between the number of familial CRC cases, and the number explained by high-risk genes, was well illustrated in 2021 by Hemminki et al. who have studied familial CRCs in the Swedish population [19]. They showed that among the 49,000 CRCs that were diagnosed in one generation, there were 7,650 patients (15.7%) who had a family history of CRC in parents or siblings; among these, only 417 (5.5%) had two or more family members (parents or siblings) affected by CRC, which could be suggestive of underlying high-risk CRC genes [19].

## Genome-wide association studies: polygenic architecture of CRC

With the beginning of the 21^st^ century and with the gap in understanding of genetic architecture of diseases left by monogenic model of inheritance, the focus shifted to complex polygenic inheritance of common low-risk variants, for which GWAS has become the most popular approach to find associations at a population level. In comparison to genome-wide linkage analysis, which is based on examining genome-wide polymorphic markers within the same family, whereby a few opportunities of recombination events have occurred, the GWAS approach examines SNPs throughout the genomes of thousands of genetically diverse individuals, allowing a higher-resolution map (Fig. 1B). However, this advantage comes with an increased probability of false-positive results if not properly accounted for [16]. The term “polygenic inheritance” refers to a non-Mendelian model of inheritance, whereby a trait is governed by the co-inheritance of 2 or more independent genes that exert additive effects [22]. With the development of GWAS, this model has gone through two major refinements, the first was the “common disease-common variant hypothesis” which correlates traits to the co-inheritance of a small number of moderate-effect loci. Then came the better-developed “infinitesimal model of inheritance” which correlates traits to co-inheritance of hundreds or thousands of common small-effect size variants [17]. As for malignancies, it is believed that most of the common cancers, such as lung and prostate cancer, are dominated by polygenic inheritance, and that most rare malignancies are dominated by monogenic inheritance. However, as the “common disease-common variant model” has not been able to explain much of the “missing heritability” of diseases [17], it is not surprising that some cancers do not follow this paradigm. Examples are ovarian cancer which is dominated by monogenic inheritance despite being a common disease, and CRC which seems to be equally dictated by monogenic and polygenic architectures [11].

GWASs have identified ~ 100 independent common low-risk loci for CRC [11, 15]. There are multiple applications of these discoveries. Firstly, they have provided an explanation of about 12% of familial relative risk of CRC which do not manifest typical Mendelian inheritance [11, 15]. Secondly, GWAS has allowed the development of polygenic risk scores (PRSs) which calculate the total risk inferred by the various risk loci harbored by individual subjects and therefore predicts their personalized risk of cancer [23]. These PRSs could be theoretically used to develop personalized cancer screening protocols. A British group has suggested adopting a personalized screening protocol for CRC based on a PRS in the top 1% of risk, which infers a 7.7-fold increased risk of developing CRC. Such an approach could result in 26% fewer subjects being eligible for screening, in comparison to the currently utilized age-based protocol, which could have positive economic implications. However, it would also decrease the number of cases detected by screening by about 6% [24]. In addition, while an elevated PRS is associated with a theoretical few-fold increased risk of a disease, it refers to a relative risk in comparison to individuals across the PRS continuum within a specific population [23, 25]. As a consequence, its utility for individual-level risk prediction is limited, as is its transferability across populations. Thus, PRSs are still considered as research tools and have not been put into clinical practice [23, 25].

Thirdly, GWASs have allowed the discovery of novel oncogenic pathways by further analysis and characterization of the function of the genes defined by candidate loci [26]. It is estimated that 20% of identified loci by GWASs include a pathogenic gene that is involved in monogenic disease inheritance [27]. In fact, GWAS might be more important to understand the somatic pathways for CRC than germline processes. A meta-analysis of GWASs conducted in 2019 has identified, in addition to the established pathways related to TGF-β/SMAD, BMP, Wnt-β-catenin, Hedgehog signaling, cell cycle, and telomere maintenance, further signals implicating Krüppel-like factors, Hippo-YAP signaling, long noncoding RNAs, somatic drivers, and supported a role of immune function [15]. However, this expanding knowledge about the polygenic architecture of CRC is challenging the current understanding of CRC biological pathways, together with the ability of developing precision therapeutics [15, 28]. Finally, as GWAS is a population-based approach designed to detect associations with common low-risk loci and not rare moderate-to-high risk genes, much of the familial CRC cases has remained unanswered despite the discoveries revealed by GWAS [13].

## Genome-wide sequencing approach: moderate-penetrance CRC genes and the new familial CRC and polyposis syndromes

A decade after the development of GWAS, whole-exome (WES) and whole-genome sequencing (WGS) approaches have been developed and led to the identification of various putative cancer predisposition variants, that could neither have been identified by linkage analysis, due to their non-Mendelian inheritance, nor by GWASs due to their rare frequencies among general populations (Fig. 1C) [29]. As for CRC, genome-wide sequencing approaches have allowed the identification of a second batch of high-to-moderate risk genes, together with a new group of familial CRC and polyposis syndromes [11]. These syndromes include polymerase proofreading-associated polyposis (PPAP) which result from an AD mutation of *POLE* or *POLD1* genes [30], *NTHL1*-associated polyposis which, similar to *MUTYH*, is involved in base-excision repair (BER) pathway, and also follows AR inheritance [31], and mismatch repair gene biallelic inactivation-related adenomatous polyposis due to mutations of *MSH3* [32] or *MLH3* [33]. Additional syndromes include *GREM1*-associated hereditary mixed polyposis syndrome (HMPS1) [34], *RNF43*-associated serrated polyposis [35], and *RPS20* mutations which is a rare cause of hereditary nonpolyposis CRC [36]. With these discoveries coming to light, it can be said nowadays that both monogenic and polygenic architectures attribute equally to the inheritance of CRC, and together they contribute about 25% of familial clustering [11].

However, as with the development of GWAS, the growing application of genome-wide sequencing and the increased amount of available genetic data, has similarly led to increased concerns regarding the utility of this knowledge. Variants of unknown significance (VUS) in known CPGs is one of these problems [37]. Lucci-Cordisco et al. [38] have reviewed current data regarding VUS of 24 CPGs included by the American College of Medical Genetics/Association for Molecular Pathology in the list of genes that should be considered for the return of incidental findings [39]. In this review, which was based on Clinvar Miner (accessed on September 14^th^, 2021) [40], the number of identified VUS for the *APC* gene, for example, was about 5100 in comparison to 1300 pathogenic variants [38]. Another problem is the increasing number of cases of CRC that are observed in families harboring germline variants in CPGs that are not generally associated with CRC, which raises the debate whether screening for CRC should be initiated in such families. *BRCA1* and *BRCA2*, which are associated with hereditary breast and ovarian cancer are the best studied in this context. Nevertheless, the debate whether pathogenic *BRCA* variants increase the risk of CRC is still ongoing [41, 42]. Other genes, raising similar debates, were summarized in a review of Valle et al. [11].

Not infrequently, genome-wide sequencing methods on familial or early-onset CRC cases, as well as within CRC families, have led to the identification of novel candidate genes, that need validation. In family-based studies, data are often analyzed using filtering approaches whereby a few affected and unaffected family members are prioritized for sequencing, to exclude variants that are not shared by affected individuals. Such an approach takes into consideration the type of the variant and available data on diverse genomic in silico databases [16]. In addition to well-established InSiGHT database with evidence-based classification of variants in high-penetrance CRC genes [4], novel approaches are emerging for evaluation of variants in novel candidate genes. An example is the familial cancer variant prioritization pipeline (FCVPP) that has been developed for detection of deleterious germline variants with potential clinical importance in cancer predisposition [43]. As well, there are increasing proposals for next-generation linkage analysis methodologies to be coupled with family-based WGS studies, instead of the filtering approach [16, 29]. Such efforts further illustrate the complementary roles of the various genome-wide approaches.

## Conclusion

To conclude, genome-wide linkage analysis, GWAS, and genome-wide sequencing approaches have allowed the current comprehensive, but not exhaustive, understanding of the genetic architecture of CRC. Nevertheless, much of the heritability of the disease has remained unexplained, and might be attributed to a broader model, including other biological and environmental modifiers.

## Data Availability

All reviewed papers were cited in the references.

## Abbreviations

CRC: Colorectal cancer
GWAS: Genome-wide association study
LS: Lynch syndrome
AD: Autosomal dominant
FAP: Familial adenomatous polyposis
HNPCC: Hereditary non-polyposis colorectal cancer
OMIM: Online Mendelian Inheritance in Man
SNPs: Single nucleotide polymorphisms
MMR: Mismatch repair
AR: Autosomal recessive
PRSs: Polygenic risk scores
WES: Whole-exome sequencing
WGS: Whole-genome sequencing
BER: Base-excision repair
HMPS1: Hereditary mixed polyposis syndrome
VUS: Variants of unknown significance
FCVPP: Familial cancer variant prioritization pipeline
